# Development and validation of polyamines metabolism-associated gene signatures to predict prognosis and immunotherapy response in lung adenocarcinoma

**DOI:** 10.3389/fimmu.2023.1070953

**Published:** 2023-06-02

**Authors:** Ning Wang, Mengyu Chai, Lingye Zhu, Jingjing Liu, Chang Yu, Xiaoying Huang

**Affiliations:** ^1^ Division of Pulmonary Medicine, the First Affiliated Hospital of Wenzhou Medical University, Key Laboratory of Heart and Lung, Wenzhou, Zhejiang, China; ^2^ Intervention Department, the First Affiliated Hospital of Wenzhou Medical University, Wenzhou, Zhejiang, China

**Keywords:** lung adenocarcinoma, polyamines metabolism, prognosis, tumor microenvironment, immunotherapy response

## Abstract

**Background:**

Polyamines metabolism is closely related to tumor development and progression, as well as tumor microenvironment (TME). In this study, we focused on exploring whether polyamines metabolism-associated genes would provide prognosis and immunotherapy response prediction in lung adenocarcinoma (LUAD).

**Methods:**

The expression profile data of polyamines metabolism-associated genes were acquired from the Cancer Genome Atlas (TCGA) database. Utilizing the least absolute shrinkage and selection operator (LASSO) algorithm, we created a risk score model according to polyamines metabolism-associated gene signatures. Meanwhile, an independent cohort (GSE72094) was employed to validate this model. Through the univariate and multivariate Cox regression analyses, the independent prognostic factors were identified. Subsequently, quantitative real-time polymerase chain reaction (qRT-PCR) was performed to detect their expression in LUAD cells. By consensus clustering analysis, polyamines metabolism-associated subgroups were determined in LUAD patients, with differential gene expression, prognosis, and immune characteristics analyses explored.

**Results:**

A total of 59 polyamines metabolism genes were collected for this study, of which 14 genes were identified for the construction of risk score model using LASSO method. High- and low- risk groups of LUAD patients in TCGA cohort were distinguished *via* this model, and high-risk group presented dismal clinical outcomes. The same prognostic prediction of this model had been also validated in GSE72094 cohort. Meanwhile, three independent prognostic factors (PSMC6, SMOX, SMS) were determined for constructing the nomogram, and they were all upregulated in LUAD cells. In addition, two distinct subgroups (C1 and C2) were identified in LUAD patients. Comparing the two subgroups, 291 differentially expressed genes (DEGs) were acquired, mainly enriching in organelle fission, nuclear division, and cell cycle. Comparing to C1 subgroup, the patients in C2 subgroup had favorable clinical outcomes, increased immune cells infiltration, and effective immunotherapy response.

**Conclusion:**

This study identified polyamines metabolism-associated gene signatures for predicting the patients’ survival, and they were also linked to immune cells infiltration and immunotherapy response in LUAD patients.

## 1 Introduction

Polyamines metabolism participate in multiple cellular processes, such as gene regulation, cell proliferation and differentiation, cell death, and immune system function (1–3). The maintenance of polyamines homeostasis requires stringent cellular regulatory process, including biosynthesis, decomposition, and transport. Previous studies have uncovered that due to increased biosynthesis and transport, and decreased catabolism, high levels of polyamines widely occur in cancer cells suggesting an important interplay between polyamines metabolism and carcinogenesis (4, 5). Polyamines metabolism is dysregulated in many tumors, which is directly associated with the development and progression of cancers. Therefore, polyamines metabolism has been considered to be an attractive target for cancer therapy.

Cancer immunotherapy, an emerging and promising treatment strategy, utilizes the enhanced antitumor effects of immune system to kill cancer cells. Recently, striking progress has been made in cancer immunotherapy, which dramatically changed the paradigm of cancer treatment (6, 7). Immunotherapy has improved cancer patients’ survival worldwide, however, most patients lack effective immune response resulting in non-sustainable disease control. Existing evidence reveals that the inadequate immune effector cells infiltration and the immunosuppressive status of immune cells in tumor microenvironment (TME) are the important mechanisms affecting the response to cancer immunotherapy. Immunosuppressive TME can hamper the antitumor actions of immune effector cells leading to the immune surveillance evasion of malignant cells, which is a vital obstacle to successful immunotherapy (8, 9). Therefore, understanding the potential regulatory mechanisms of the immune status in TME is critical for improving the efficacy of immunotherapy.

According to research findings, polyamines biosynthetic enzymes were upregulated in tumor tissues, and the elevated spermine and spermidine promoted tumor growth and correlated with immunosuppressive status of TME (10). In addition, many immune cell types in TME, including myeloid-derived suppressor cells (MDSCs), dendritic cells and M2 macrophages, translated from an immune-active to an immune-suppressive state affecting antitumor immunity owing to polyamines metabolic disorders (11). Considering the dependence of tumor cells on polyamines and the crucial biological function of polyamines in immune cells, targeting polyamines metabolic pathways are expected to be an important cancer therapeutic strategy. In particular, it will be extremely beneficial to explore biomarkers based on polyamines metabolism that can predict the response to immunotherapy in tumor patients.

Lung adenocarcinoma (LUAD), the most common subtype of lung cancer, has a dismal prognosis, with relatively high mortality rate in malignant tumor patients (12, 13). Despite advancements in traditional cancer treatments over the last few decades, there has limited improvements in patients’ survival outcomes. Immunotherapy, especially immune checkpoint inhibitors (ICIs), has been considered as an important therapeutic option for LUAD patients with favorable improvement in survival (14, 15). Currently, PD-L1 expression is still considered as a biomarker for predicting the patients who will benefit from immune checkpoint blockade (ICB) therapy. However, many patients do not present a good response. It is, therefore, meaningful to identify the new biomarkers precisely predicting immunotherapy response.

In this study, we investigated the expression pattern of polyamines metabolism genes in LUAD, and screened out the significant gene signatures based on machine learning method to develop a risk score model, which could predict patients’ survival. This model also was validated in another independent cohort, and the expression of prognostic factors were detect using qRT-PCR. Furthermore, the subgroups of LUAD patients classified by these significant gene signatures had different immune cells infiltration levels and immunotherapy response. This is the first study to explore the role of polyamines metabolism-related gene signatures in LUAD patients from the perspective of patients’ prognosis and immunotherapy response.

## 2 Materials and methods

### 2.1 Data acquisition

RNA-sequencing expression profile data and matched clinical information of LUAD samples were collected from The Cancer Genome Atlas (TCGA) database (https://portal.gdc.cancer.gov/). Validation dataset (GSE72094) was obtained from Gene Expression Omnibus (GEO) database (http://www.ncbi.nlm.nih.gov/geo/). After standardized processing, the data was conducted for further analysis using R software. A total of 59 polyamines metabolism-associated genes were collected from MSigDB database (http://www.gsea-msigdb.org/). By searching the keyword “polyamines”, the gene sets “REACTOME_METABOLISM_OF_ POLYAMINES” were discovered. The detail gene information is listed in **Supplementary Materials**.

### 2.2 Gene expression and protein–protein interaction network

We first investigated the expression levels of 59 polyamines metabolism-associated genes between tumor and normal tissues. Furthermore, PPI network was established based on STRING platform (https://string-db.org/) to analyze their interconnections (16).

### 2.3 Identification of polyamines metabolism-associated gene signatures for risk score model in LUAD

Based on LASSO algorithm using “glmnet” R package (17), the significant polyamines metabolism gene signatures were explored to construct the risk score model. Application of the optimal cut-off value, LUAD patients were distinguished into two groups: high- and low- risk groups, for further prognosis analysis. In addition, we verified the predictive ability of risk score model *via* the external independent dataset (GSE72094).

### 2.4 Prognosis analysis

We first respectively compared the prognosis between high- and low-risk groups in TCGA and GSE72094 LUAD cohorts using the “survival” package. Furthermore, the univariate and multivariate Cox regression analyses were carried out to recognize the independent prognostic factors from polyamines metabolism-associated genes in LUAD. Subsequently, these factors were used for establishing a prognostic nomogram *via* the “rms” package, which could be employed for survival (1-, 3-, 5-year) prediction of LUAD patients.

### 2.5 Receiver operating characteristic curve analysis

The predictive performance of gene expression was judged using ROC curve analysis conducted by the “pROC” package. The area under the curve (AUC) value was calculated for quantitative analysis according to previous methods (18).

### 2.6 Cell culture and qRT-PCR

Human bronchial epithelial cells (BEAS-2B) and human LUAD cells (H1975 and H2009) were acquired from the American Type Culture Collection (ATCC, United States). All cells were conventionally cultured and the total RNA was collected according to commercial kit methods. After reverse transcription *via* Hiscript III All-in-one RT Super mix Perfect for qPCR kit (Vazyme, China), qRT-PCR was conducted using Taq Pro Universal SYBR qPCR Master Mix kit (Vazyme, China). GAPDH served as an endogenous control. Primers were synthesized from Sangon Biotech (Shanghai, China) and the detail information of the sequences were provided in **Supplementary Materials**.

### 2.7 Recognition of the subgroups in LUAD patients *via* consensus clustering analysis

The consensus clustering analysis was applied to recognize the polyamines metabolism-associated subgroups in LUAD patients, which was conducted *via* the “ConsensusClusterPlus” package.

### 2.8 Differential gene analysis

Differential gene analysis was carried out to recognize the DEGs between the different subgroups *via* the “Limma” package (19) based on the criteria of adjusted *P* < 0.05 and Fold Change > 2. Then, volcano plot and heatmap were applied to display these differential genes.

### 2.9 Function enrichment analysis

GO enrichment, including the biological process (BP), cellular component (CC), and molecular function (MF), and KEGG enrichment of these significant polyamines metabolism genes were conducted. Additionally, function enrichment analyses of the upregulated DEGs in subgroups were carried out *via* the “ggplot2” and “ClusterProfiler” packages.

### 2.10 Clinicopathological features analysis

The association between the clinicopathological features such as age, TNM stage, and survival status in different subgroups of LUAD patients was described using Sankey diagram *via* the “ggalluval” package. Moreover, overall survival analysis was performed in different subgroups of LUAD patients.

### 2.11 Somatic mutation analysis

Somatic mutation analysis in different subgroups were performed *via* the “Maftools” package. The whole somatic mutation landscape was displayed using waterfall plots.

### 2.12 Immune characteristics analysis

We first assessed the immune status of TME, including Immune Score, Stromal Score and ESTIMATE Score in different subgroups *via* ‘‘estiate” package. Furthermore, the immune cells infiltration status was investigated using MCP-counter (20) and xCell (21) algorithms performed by the “immunedeconv” package. Finally, we assessed the immunotherapy response to ICB in different subgroups by means of Tumor Immune Dysfunction and Exclusion (TIDE) algorithm (22).

## 3 Results

### 3.1 The expression of polyamines metabolism-related genes in LUAD

**Figure 1:**
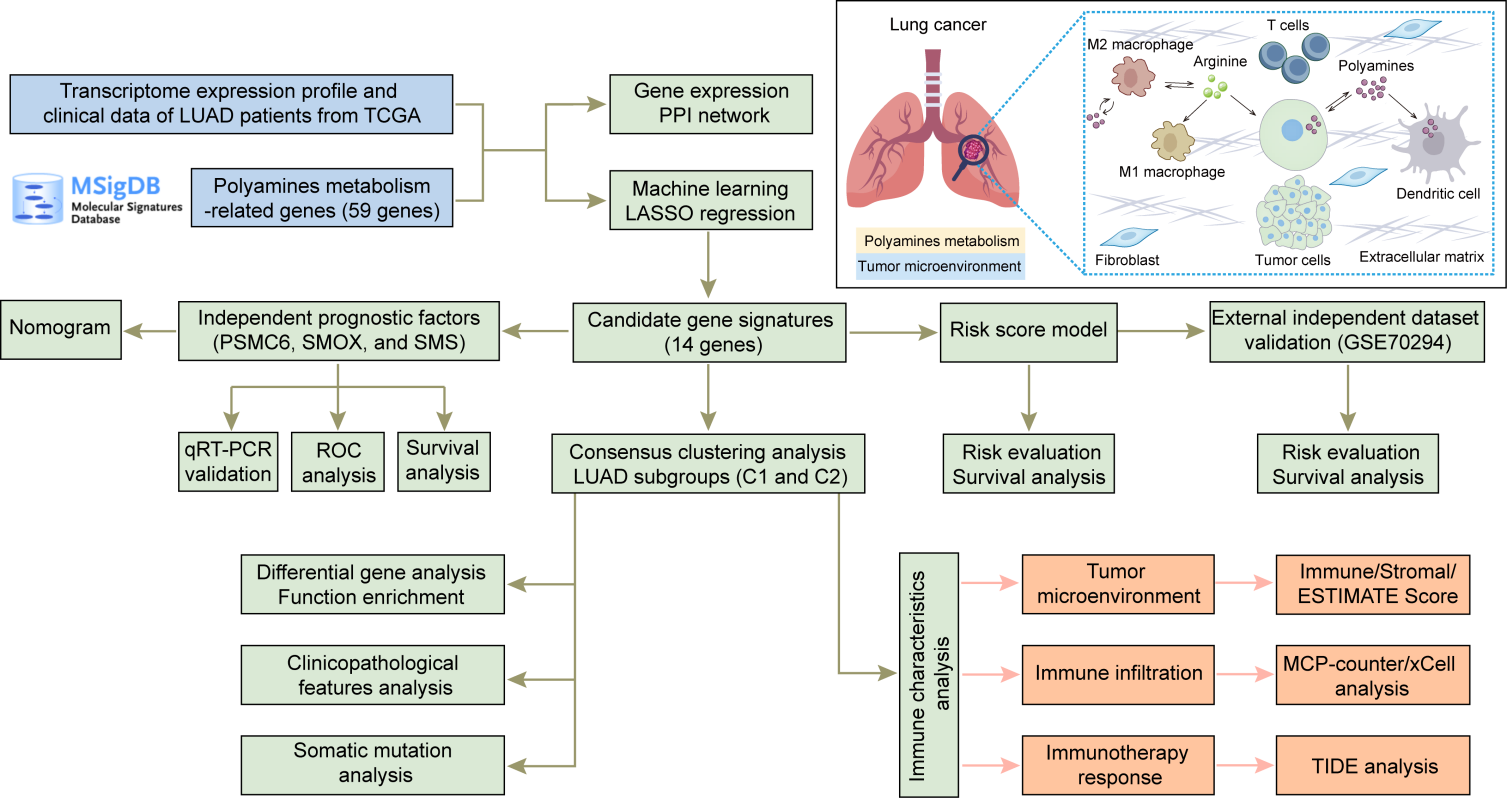
Flow diagram of the overall study and the pattern diagram of polyamines metabolism in tumor microenvironment.

**Figure 2:**
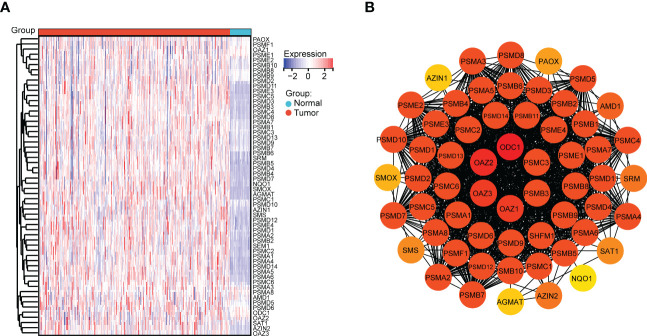
Gene expression and protein–protein interaction (PPI) network. **(A)** Heatmap depicting the expression of polyamines metabolism-associated genes between tumor and normal tissues based on TCGA database. Different colors represent the trend of gene expression in different tissues. **(B)** PPI network among polyamines metabolism-associated genes. Different depth color represents the degree score of the protein.

The entire study is conducted according to the summarizing flow chart (**Figure 1**). A total of 59 polyamines metabolism-related genes were collected from MSigDB database. The expression of these polyamines metabolism genes in normal and LUAD samples were first explored. As shown in **Figure 2A**, most of polyamines metabolism genes were overexpressed in LUAD samples. The STRING platform was employed to construct PPI network, investigating the connections among these polyamines metabolism-associated genes (**Figure 2B**).

### 3.2 Construction and validation of polyamines metabolism-associated risk score model in LUAD

**Figure 3:**
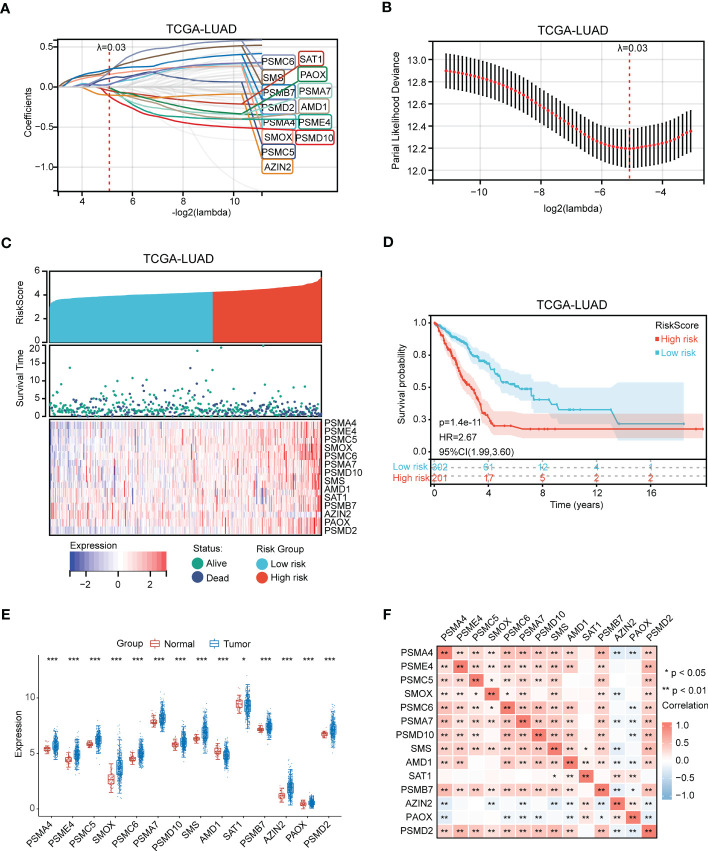
Identification of polyamines metabolism-associated gene signatures for risk score model. **(A)** Screening of the polyamines metabolism-associated gene signatures for risk score model using the LASSO algorithm. **(B)** The best log (Lambda) value in the model. **(C)** Heatmap depicting genes expression distribution and survival status in LUAD patients. **(D)** Kaplan-Meier plot depicting overall survival of high- and low- risk LUAD patients. **(E)** Box plot depicting the expression of gene signatures in risk score model. **(F)** Heatmap depicting the correlation among these gene signatures. Statistical analysis: *P < 0.05, **P < 0.01, and ***P < 0.001.

The significant gene signatures were further narrowed down from 59 polyamines metabolism-associated genes using LASSO regression machine learning method in LUAD. The result showed a total of 14 significant gene signatures were eventually determined to establish the risk score model on the basis of the optimal λ value 0.02949 (**Figures 3A, B**). The risk score was calculated following equation: Risk Score = 0.036*PSMA4 + (-0.003)*PSME4 + 0.109*PSMC5 + 0.157*SMOX + 0.188*PSMC6 + (-0.011)*PSMA7 + (-0.117)*PSMD10 + 0.206*SMS + (-0.015)*AMD1 + (-0.044)*SAT1 + 0.224*PSMB7 + (-0.101)*AZIN2 + (-0.003)*PAOX + 0.028*PSMD2. According to the optimal cut-off value (4.26), the patients were divided into two groups with high and low risk. As shown in **Figure 3C**, the LUAD patients with high-risk scores presented high risk of death. Prognosis analysis indicated that high-risk patients had poor prognosis (*P* = 1.4 e-11, HR = 2.67, **Figure 3D**). Moreover, we discovered that except for high expression of AMD1 and SAT1 in normal samples, other genes (PSMA4, PSME4, PSMC5, SMOX, PSMC6, PSMA7, PSMD10, SMS, PSMB7, AZIN2, PAOX, PSMD2) were all upregulated in tumor samples (**Figure 3E**). Gene expression correlation analysis revealed that there presented a positive correlation among these genes (PSMA, PSME4, PSMC5, SMOX, PSMC6, PSMA7, PSMD10, SMS, AMD1, PSMB7, and PSMD2) (**Figure 3F**).

**Figure 4:**
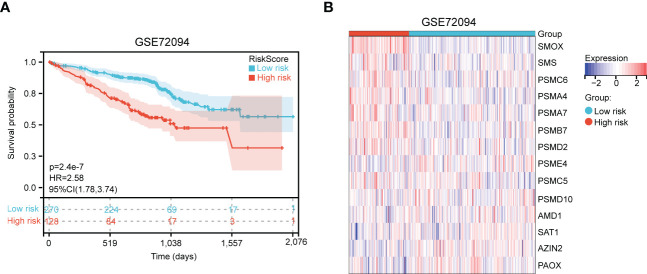
Validation of polyamines metabolism-associated risk score model in GSE72094 cohort. **(A)** Kaplan-Meier plot depicting overall survival of high- and low- risk LUAD patients in the independent cohort. **(B)** Heatmap depicting the expression of these gene signatures in risk score model in the independent cohort.

Next, an independent cohort (GSE72094) was utilized to validate the predictive effectiveness of risk score model in the prognosis of LUAD patients. Based on the optimal cut-off value (4.28), high and low risk groups were distinguished. We also discovered that high-risk patients had poor prognosis (**Figure 4A**). Finally, the expression of 14 genes in high- and low- risk groups were exhibited *via* heatmap (**Figure 4B**).

### 3.3 Function enrichment analysis of the 14 gene signatures in risk score model

**Figure 5:**
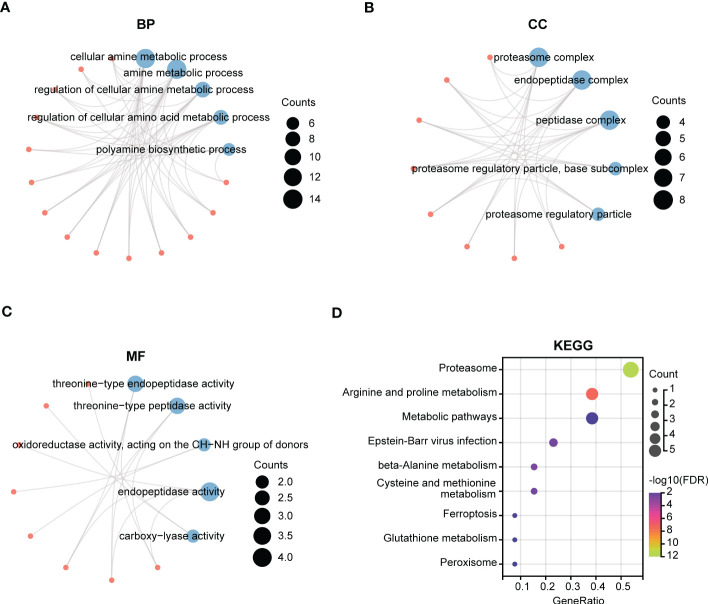
Function enrichment analysis of polyamines metabolism-associated gene signatures. **(A-C)** The top5 pathways of **(A)** BP (biological process), **(B)** CC (cellular component), and **(C)** MF (molecular function) in GO enrichment analysis of polyamines metabolism-associated gene signatures. **(D)** KEGG enrichment analysis of these gene signatures. The size of the circle means the number of enriched genes, and the larger the number, the larger the circle.

We next performed GO and KEGG enrichment analyses of the 14 gene signatures in risk score model respectively. As shown in **Figures 5A–C**, the Top5 enrichment results of BP, CC, and MF were exhibited. Moreover, KEGG analysis results indicated that these genes were primarily enriched in proteasome, arginine and proline metabolism, and metabolic pathways (**Figure 5D**).

### 3.4 Recognition of the independent prognostic factors and construction of the nomogram

**Figure 6:**
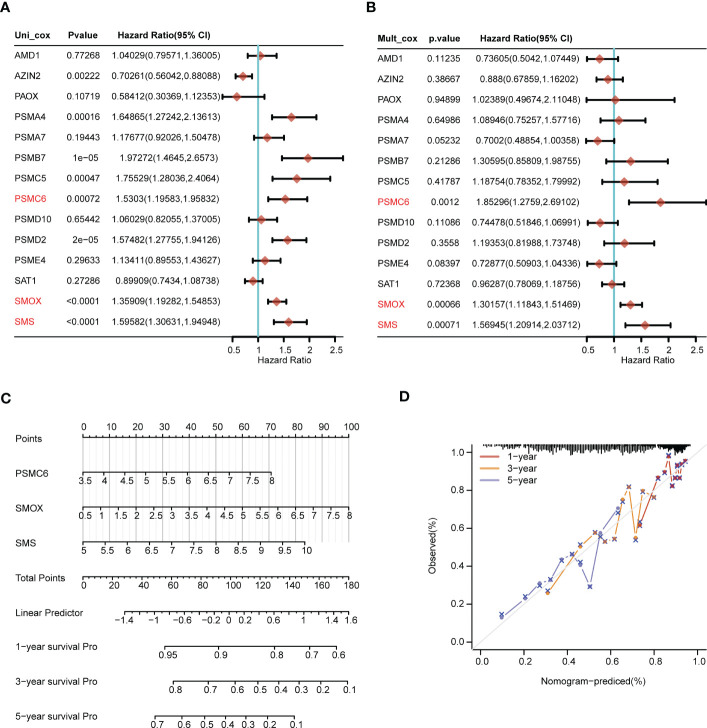
Construction of the nomogram predicting patients’ survival in LUAD. **(A)** The univariate and **(B)** multivariate Cox regression analyses of polyamines metabolism-associated gene signatures for exploring the independent prognostic factors in LUAD. HR more than 1 indicates the risky gene, and HR less than 1 indicates the protective gene. **(C)** Construction of the nomogram for survival prediction (1-, 3-, 5-year) of LUAD patients. **(D)** Calibration curve of the nomogram.

Through univariate Cox regression analysis, we discovered that AZIN2, PSMA4, PSMB7, PSMC5, PSMC6, PSMD2, SMOX, and SMS were significantly correlated with patients’ OS. The hazard ratio of AZIN2 (HR < 1, *P* < 0.05) favored patients’ prognosis, and the other genes, including PSMA4, PSMB7, PSMC5, PSMC6, PSMD2, SMOX, and SMS, were all risk factors (HR > 1, *P* < 0.05) (**Figure 6A**). We further conducted the multivariate Cox regression analysis, and discovered that PSMC6, SMOX, and SMS were also significantly correlated with OS, all serving as risk factors (HR > 1, *P* < 0.05) (**Figure 6B**). Thus, comprehensive analyses revealed that PSMC6, SMOX, and SMS could be recognized as independent prognostic factors. Based on the three factors, we constructed a nomogram, providing a certain predictive effect of clinical prognosis (**Figures 6C, D**).

**Figure 7:**
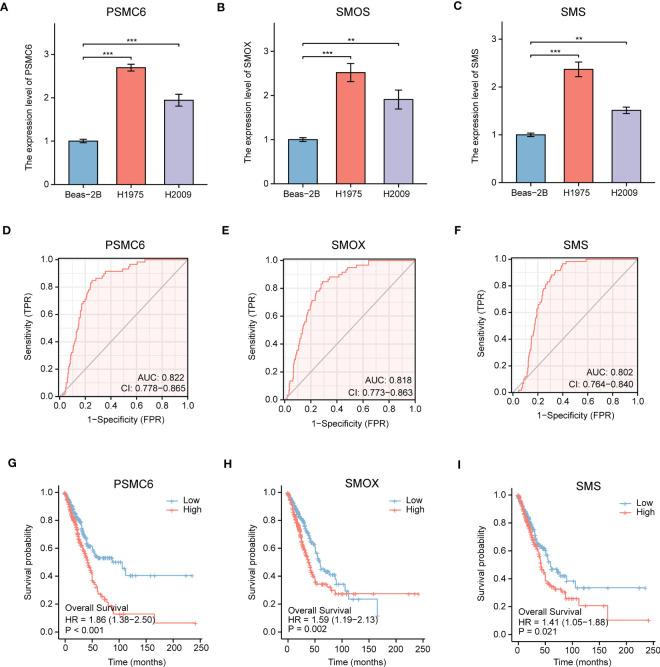
Comprehensive analysis of gene expression, diagnostic value, and prognosis analysis of the independent factors (PSMC6, SMOX, SMS). **(A-C)** The gene expression levels of **(A)** PSMC6, **(B)** SMOX, and **(C)** SMS in normal lung cells (Beas-2B) and LUAD cells (H1975 and H2009) by qRT-PCR. GAPDH serves as an endogenous control. **(D-F)** ROC curve analyses for **(D)** PSMC6, **(E)** SMOX, and **(F)** SMS expression in LUAD. The AUC value represents the predictive performance. **(G-I)** Kaplan-Meier plot depicting the predictive role of **(G)** PSMC6, **(H)** SMOX, and **(I)** SMS expression for patients’ survival. Statistical analysis: **P < 0.01 and ***P < 0.001.

Next, we explored the expression levels of PSMC6, SMOX, and SMS in LUAD cell lines, and their diagnostic and prognostic values in LUAD patients, respectively. PSMC6, SMOX, and SMS were all upregulated in LUAD cell lines (H1975 and H2009), comparing to normal bronchial epithelial cells (BEAS-2B) (**Figures 7A–C**). ROC analysis revealed that the AUC values of PSMC6, SMOX, and SMS were 0.822, 0.818, and 0.802, respectively, suggesting they had certain diagnostic values (**Figures 7D–F**). Finally, survival analysis revealed that the patients with high expression of PSMC6, SMOX, and SMS presented poor prognosis (**Figures 7G–I**).

### 3.5 Consensus clustering analysis recognized polyamines metabolism-associated subgroups in LUAD

**Figure 8:**
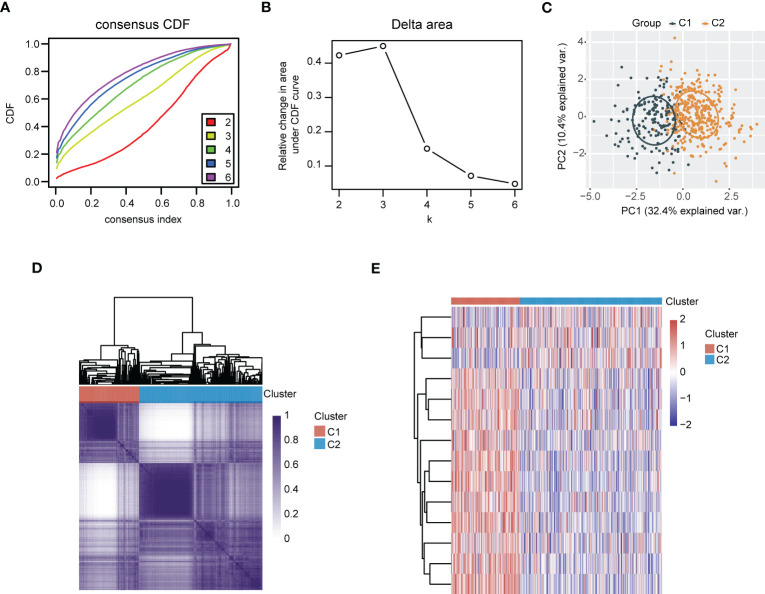
Recognition of polyamines metabolism-associated subgroups in LUAD patients through consensus clustering analysis. **(A)** Consensus clustering of the cumulative distribution function (CDF) curve. **(B)** The relative change in area under CDF curve. **(C)** Principal component analysis (PCA) of C1 and C2 subgroups. **(D)** Heatmap of consensus clustering (k = 2). **(E)** Heatmap depicting the expression of gene signatures in C1 and C2 subgroups of LUAD patients.

**Figure 9:**
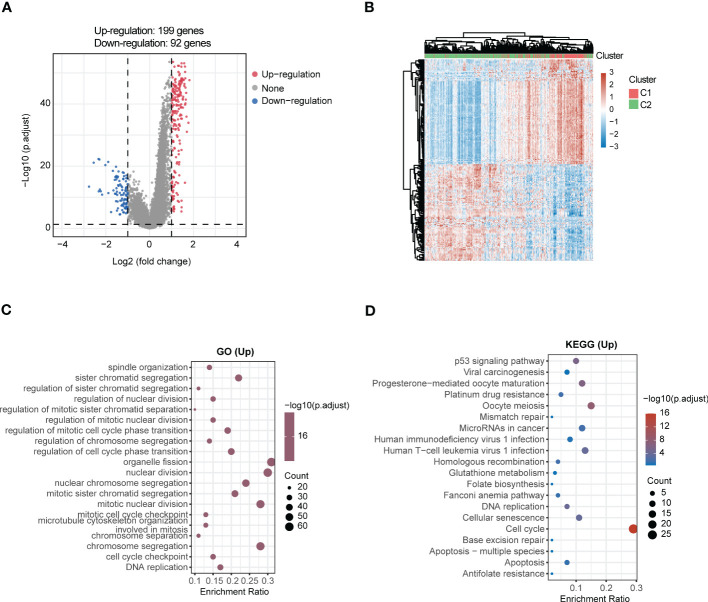
Comprehensive analysis of DEGs and function enrichment between the two subgroups in LUAD. **(A)** Volcano plot depicting DEGs between the two subgroups. Red means the upregulated genes, blue means the downregulated genes, and grey means not significant. **(B)** Heatmap of DEGs in the two subgroups. **(C)** GO and **(D)** KEGG enrichment analysis of the upregulated DEGs.

LUAD samples were divided into two different subgroups (C1 and C2) relying on consensus clustering analysis (**Figures 8A–D**). Subsequently, the expression of 14 genes between the two subgroups were displayed (**Figure 8E**). In addition, a total of 291 DEGs were screened out by comparing the two different subgroups, of which 199 DEGs were upregulated and 92 DEGs were downregulated (**Figure 9A**). The detail gene information is listed in **Supplementary Materials**. The expression of DEGs were displayed *via* heatmap (**Figure 9B**). Finally, function enrichment analyses revealed that GO analysis of upregulated DEGs mainly converged at organelle fission, nuclear division, mitotic nuclear division, and chromosome segregation (**Figure 9C**). The KEGG analysis of upregulated DEGs mainly converged at cell cycle (**Figure 9D**).

### 3.6 Comprehensive analysis of clinicopathological features, somatic mutation and immune landscape in the subgroups

**Figure 10:**
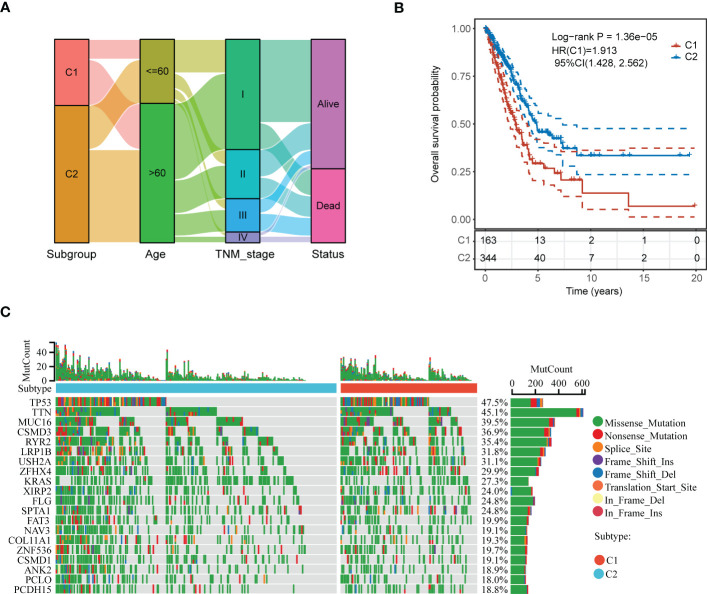
Comprehensive analysis of clinicopathological features and somatic mutation between the two subgroups of LUAD patients. **(A)** Sankey diagram depicting the different clinicopathological features in C1 and C2 subgroups of LUAD patients. **(B)** Kaplan-Meier plot depicting overall survival in C1 and C2 subgroups of LUAD patients. **(C)** Waterfall plot depicting the somatic mutation feature in C1 and C2 subgroups.

We first analyzed the different clinicopathological features such as age, TNM stage, and survival status in C1 and C2 subgroups using Sankey diagram (**Figure 10A**). Kaplan-Meier plots indicated that the patients in C1 subgroup presented a dismal prognosis in OS, comparing to C2 subgroup (**Figure 10B**). In addition, we discovered that there presented different genes mutation between C1 and C2 subgroups such as TP53, TTN, MUC16, and so on (**Figure 10C**).

**Figure 11:**
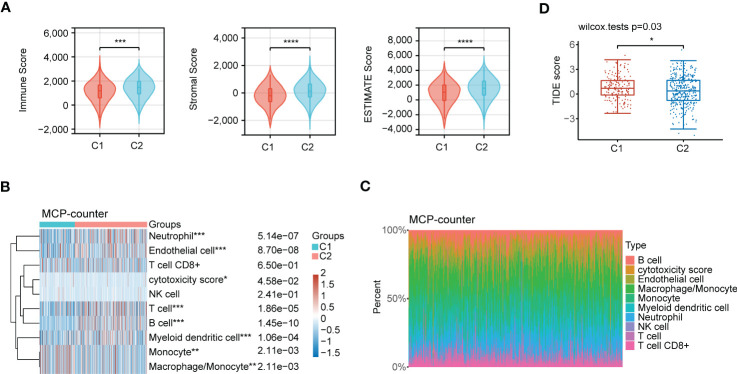
Immune characteristics analysis in the subgroups of LUAD patients. **(A)** Comprehensive analysis of the immune status in TME, including Immune Score, Stromal Score and ESTIMATE Score between C1 and C2 subgroups. **(B)** Heatmap depicting immune cells infiltration in C1 and C2 subgroups using MCP-counter algorithm. **(C)** Stacking plot depicting immune cell abundance in TME using MCP-counter algorithm. **(D)** The prediction of immunotherapy response between the two subgroups according to the TIDE score. Statistical analysis: *P < 0.05, **P < 0.01, ***P < 0.001, and ****P < 0.0001.

Accumulating evidence reveals that polyamines metabolism is correlated with tumor immunosuppressive microenvironment and promotes tumor growth. In this research, we first investigated the composition of the tumor microenvironment between C1 and C2 subgroups. The result indicated that C2 subgroup presented a higher immune infiltration status than C1 subgroup (**Figure 11A**). We next assessed the infiltration levels of immune cells between C1 and C2 subgroups utilizing MCP-counter algorithm. The samples in C2 subgroup had significantly higher immune cells infiltration, such as neutrophil, T cell, B cell, and myeloid dendritic cell than those in C1 subgroup (**Figures 11B, C**). In addition, we performed another algorithm (xCell) analysis, and also noted that there were high infiltration levels of most of immune cells in C2 subgroup (**Figures S1A, B**). Finally, TIDE algorithm was applied to assess the immunotherapy response to ICB between C1 and C2 subgroups. High TIDE score reveals poor response to ICB and short survival. As shown in **Figure 11D**, the patients in C2 subgroup had relatively satisfied treatment response to immunotherapy.

## 4 Discussion

Metabolic reprogramming has been widely considered to be a key mechanism in tumorigenesis and progression (23, 24). Metabolic reprogramming, an important hallmark of tumor cells, accelerates cell proliferation by regulating metabolism-related processes. Polyamines are not only involved in gene regulation, but also in a series of signal transduction processes, exerting a crucial role in cell proliferation and survival. Notably, dysregulation of polyamines metabolism leads to the elevation of polyamines in cancers, maintaining the growth and progression of tumor cells (25). Previous research have shown that polyamines dysregulation contributes to the progression of helicobacter pylori-induced gastric cancer (26). In addition, the study of potential regulatory mechanism of polyamines metabolism exerts the vital means for understanding the tumor evolving process (27). Therefore, polyamines metabolic pathway is a promising target for anti-tumor treatment.

Here, we discovered that most of polyamines metabolism-related genes were overexpressed in LUAD samples. In order to clarify the association between polyamines metabolism and LUAD patients’ prognosis, we developed a risk score model using polyamines metabolism genes *via* machine learning method, predicting the different prognosis of the high- and low- risk patients. Next, we further validated the predictive effects of this model using an independent cohort, and discovered that this model could also precisely predict the patients’ prognosis. These results suggested that polyamines metabolism genes-constructed model presented a good prediction efficiency. Considering the heterogeneity among LUAD patients, subgroup analysis was a good strategy for in-depth study. Based on these genes in the model, the LUAD patients were divided into two distinct subgroups by means of consensus clustering method. The two subgroups also presented different prognosis, which verified again the predicted effects of these gene signatures. To sum up, polyamines metabolism-related gene signatures had significant predictive effects on the patients’ prognosis.

In recent years, immunotherapy has been the fastest-growing antitumor therapy. Despite great advances have been made in the application of immune checkpoint blockade therapy in multiple cancers, only a minority of patients have durable responses (28). A more in-depth investigation of complex immune landscape in TME is crucial for identifying the influencing factors in immunotherapy response. New research has found that tumor metabolism not only exerts a vital role in maintaining tumor survival, but also influences immune cells function by releasing metabolites in TME. Metabolic competition between tumor cells and immune cells, limiting efficient supply of nutrients to immune cells, impedes the antitumor function of immune cells (29, 30). Thus, metabolic changes in TME have been recognized as one of the important influences on the effects of tumor immunotherapy. In the process of tumor development, most of the cellular components in TME undergo metabolic reprogramming, and cells metabolic state has important implications for anti-tumor immunotherapy (31). Recent researches have indicated that polyamine metabolism-associated pathways have a profound impact on tumor microenvironment, and perform a crucial role in immune surveillance (32). In addition, polyamine metabolism is involved in anti-tumor immunity, and the assessment of polyamine levels can be utilized to predict the response to immunotherapy (33).

Immune cells infiltration may be required for the response to ICB therapy, and immune cells status in TME also perform a critical role in tumor immunotherapy (34). The research on immune response mechanisms of “cold” tumors and “hot” tumors will contribute to more accurately recognizing the patient groups that benefit from immunotherapy. Polyamines participate in regulating antitumor immune responses, and high polyamines levels are associated with the immunosuppressive effects (35). Through reducing the levels of polyamines can attenuate the proliferation of tumor cells, while improve the immunogenicity of “cold” tumors. In our study, the patients in C2 subgroup possessed relatively high immune cells infiltration. In addition, they also had relatively satisfied immunotherapy response according to TIDE score. These results indicated that polyamines metabolism-associated gene signatures could be utilized to predict the immunotherapy response. Therefore, tumor cells relying on polyamines and the important physiological roles of polyamines in various immune cell types suggests that targeting polyamines metabolic pathways may become a crucial focus for improving the efficacy of immunotherapy.

In conclusion, this study first developed and validated the important biological function of polyamines metabolism gene signatures for judging prognosis in LUAD patients. More importantly, the candidate genes (PSMC6, SMOX, and SMS) were identified as independent prognostic factors, constructing the nomogram to predict the patients’ prognosis. In addition, we determined the expression levels of PSMC6, SMOX, and SMS in LUAD cell lines, and explored their diagnostic and prognostic values in LUAD patients using ROC and survival analyses. We also highlighted the correlation of polyamines metabolism-associated subgroups in LUAD patients with immune cells infiltration in TME, and the immunotherapy response. These observations portended that targeting the polyamines metabolic pathway may become a promising therapeutic strategy for LUAD patients.

## Data availability statement

Publicly available datasets were analyzed in this study. This data can be found here: LUAD data was collected from The Cancer Genome Atlas (TCGA) database (https://portal.gdc.cancer.gov/); GSE72094 dataset was acquired from GEO database (http://www.ncbi.nlm.nih.gov/geo/).

## Author contributions

XH and CY contributed to the design, supervision, and acquiring funding of this work. NW and LZ performed data collection, analysis, and experiments. MC and JL participated in data analysis and figure generation. NW wrote the manuscript. All authors contributed to the article and approved the submitted version.

## Supplementary material

Supplementary Figure 1 Analysis of immune cells infiltration between C1 and C2 subgroups of LUAD patients using xCell algorithm. **(A)** Heatmap depicting immune cells infiltration in C1 and C2 subgroups using xCell algorithm. **(B)** Stacking plot depicting immune cell abundance in TME using xCell algorithm.
