# Supplementary figures and images for: Development and validation of polyamines metabolism-associated gene signatures to predict prognosis and immunotherapy response in lung adenocarcinoma
Source: Front Immunol. 2023 Jun 2;14:1070953. doi: 10.3389/fimmu.2023.1070953 (PMC10272553; doi:10.3389/fimmu.2023.1070953)

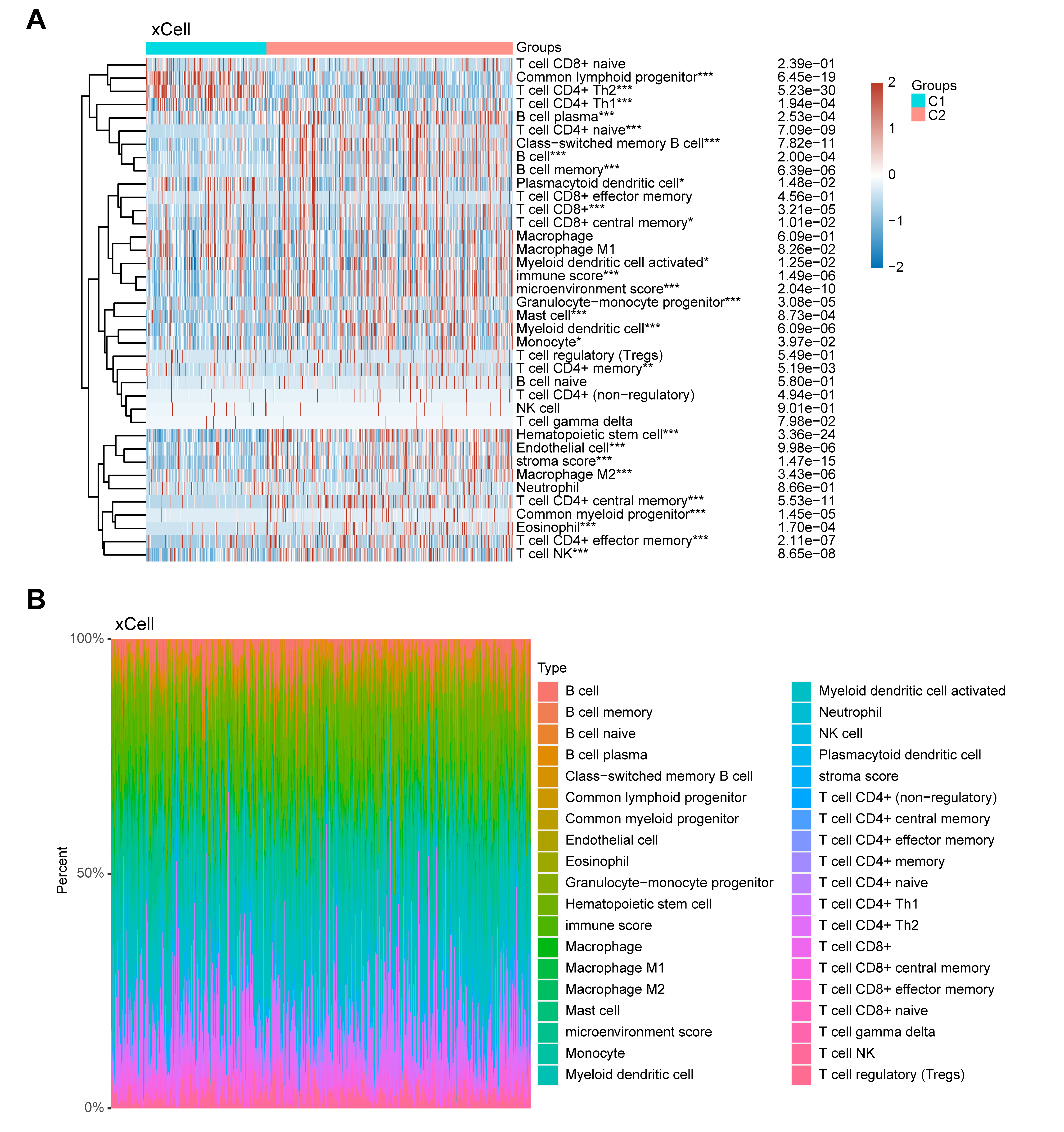

Supplement: Supplementary Figure 1 — Analysis of immune cells infiltration between C1 and C2 subgroups of LUAD patients using xCell algorithm. (A) Heatmap depicting immune cells infiltration in C1 and C2 subgroups using xCell algorithm. (B) Stacking plot depicting immune cell abundance in TME using xCell algorithm. [file Image_1.tif]
